# Granulocytic myeloid-derived suppressor cells promote angiogenesis in the context of multiple myeloma

**DOI:** 10.18632/oncotarget.9270

**Published:** 2016-05-10

**Authors:** Marilène Binsfeld, Joséphine Muller, Virginie Lamour, Kim De Veirman, Hendrik De Raeve, Akeila Bellahcène, Els Van Valckenborgh, Frédéric Baron, Yves Beguin, Jo Caers, Roy Heusschen

**Affiliations:** ^1^ Laboratory of Hematology, GIGA-Research, University of Liège, B-4000 Liège, Belgium; ^2^ Metastasis Research Laboratory, GIGA-Research, University of Liège, B-4000 Liège, Belgium; ^3^ Department of Hematology and Immunology, Myeloma Center Brussels, Vrije Universiteit Brussel, B-1090 Brussels, Belgium; ^4^ Department of Pathology, OLV Ziekenhuis Aalst, B-9300 Aalst, Belgium

**Keywords:** multiple myeloma, myeloid-derived suppressor cells, angiogenesis, pro-angiogenic, osteoclasts

## Abstract

Multiple myeloma (MM) is a plasma cell malignancy characterized by the accumulation of tumor cells in the bone marrow (BM) and is associated with immunosuppression, angiogenesis and osteolysis. Myeloid-derived suppressor cells (MDSCs) represent a heterogeneous population of immature, immunosuppressive myeloid cells that promote tumor progression through different mechanisms.

In this work, we studied the contribution of MDSC subsets to different disease-promoting aspects in MM. We observed an expansion of polymorphonuclear/granulocytic (PMN-)MDSCs in two immunocompetent murine MM models, while this was not observed for monocytic (MO-)MDSCs. Both MDSC subpopulations from MM-bearing mice were immunosuppressive, but PMN-MDSCs displayed a higher suppressive potential. Soluble factors secreted by MM cells increased the viability of MDSCs, whereas the presence of MDSCs did not affect the proliferation of MM cells *in vitro* or *in vivo*. Interestingly, we observed a pro-angiogenic effect of PMN-MDSCs in the context of MM using the chick chorioallantoic membrane assay. Consistently, MM-derived PMN-MDSCs showed an up-regulation of angiogenesis-related factors and reduced PMN-MDSC levels were associated with less angiogenesis *in vivo*. Finally, we identified MO-MDSCs as osteoclast precursors.

These results suggest that MDSC subpopulations play diverging roles in MM. We show for the first time that PMN-MDSCs exert a pro-angiogenic role in MM.

## INTRODUCTION

The plasma cell malignancy multiple myeloma (MM) is the second most frequent hematological malignancy and is characterized by the accumulation of monoclonal tumor cells in the bone marrow (BM). The BM invasion causes osteolytic bone destruction, giving rise to severe bone pain and pathological fractures in the vast majority of MM patients [1]. MM progression is facilitated by several disease-associated mechanisms, including angiogenesis [2] and immunosuppression [3]. The immune system is actively suppressed by MM cells through the secretion of suppressive factors and the recruitment of immune suppressive cells such as regulatory T cells (Tregs) [3] and myeloid-derived suppressor cells (MDSCs), a heterogeneous population of immature myeloid cells.

MDSCs have been shown to accumulate in several pathological conditions including cancer. In mice, they are characterized by the co-expression of the CD11b and Gr1 surface molecules [4]. Two different MDSC subpopulations with distinct morphological, molecular and functional properties can be distinguished: a polymorphonuclear or granulocytic population (PMN-MDSCs), resembling immature neutrophils, and a mononuclear or monocytic population (MO-MDSCs). On a phenotypical level, they can be distinguished by the differential expression of the Ly6G and Ly6C surface molecules, both recognized by the anti-Gr1 antibody. PMN-MDSCs present a CD11b^+^Ly6G^+^Ly6C^int^ phenotype, whereas MO-MDSCs are CD11b^+^Ly6G^−^Ly6C^high^. Both PMN- and MO-MDSCs suppress antigen-specific T-cell responses, even though they use different mechanisms [5, 6]. Interestingly, in a majority of murine tumor models, a preferential expansion of PMN-MDSCs has been observed [6]. MDSCs contribute to tumor progression not only by suppressing immune responses, but also by promoting tumor invasion and metastasis as well as tumor angiogenesis [7].

A pro-angiogenic role of total CD11b^+^Gr1^+^ MDSCs has been established in several murine models. In colorectal cancer and Lewis lung carcinoma (LLC), MDSCs promote angiogenesis through the production of matrix metalloproteinase 9 (MMP-9) and by the acquisition of endothelial cell properties in the tumor microenvironment [8]. Treatment with granulocyte-colony stimulating factor (G-CSF) promotes LLC and squamous carcinoma growth by promoting tumor angiogenesis, mediated by an increase in circulating endothelial progenitor cells and total MDSCs [9]. Moreover, in canine mammary cancer, an accumulation of interleukin (IL)-28-secreting MDSCs promotes the pro-angiogenic phenotype of tumor cells [10]. Regarding the involvement of MDSC subpopulations, a pro-angiogenic role of MO-MDSCs has been shown in murine lung, melanoma and prostate tumors, regulated by macrophage colony stimulating factor (M-CSF) signaling. Moreover, MO-MDSCs could contribute to a compensatory mechanism involved in resistance to antiangiogenic therapy by modulating the expression of MMP-9 [11].

In contrast to the murine MDSC phenotype, specific markers for human MDSCs are less well defined. Generally, human MDSCs are described as CD11b^+^CD33^+^HLA-DR^low/−^. As in mice, granulocytic and monocytic subpopulations can be distinguished, suggested to be CD14^−^CD15^+^ or CD14^+^, respectively [12]. In MM patients, a first report described increased CD14^+^HLA-DR^low/−^ MO-MDSC levels in the peripheral blood at diagnosis [13]. Conversely, other reports described increased levels of PMN-MDSCs in the peripheral blood and BM of MM patients [14–16]. *In vitro*, MDSCs isolated from MM patients are able to suppress T-cell responses [15, 16], induce Treg cells [16] and promote MM cell growth while MM cells induce MDSC development, indicating bidirectional interactions between MDSCs and myeloma cells [15]. In murine MM models, an early and transient accumulation of PMN-MDSCs has been observed in the BM, which is critical for MM progression by inhibiting T-cell responses [14]. In the 5T2 and 5T33 murine MM models, a decrease of overall BM MDSC levels has been noted along with MM progression. However, in that report, an expansion of the monocytic fraction has been described within CD11b^+^ BM cells. Both MDSC subpopulations isolated from these mice were able to suppress antigen-specific T-cell proliferation [17]. Finally, MDSCs (CD11b^+^Gr1^+^) could serve as osteoclast progenitors in the 5TGM1 model, implicating these cells in MM-related bone disease [18].

Taken together, the data concerning the accumulation of MDSCs in MM are conflicting or fail to discern the two different MDSC subpopulations. In this work, we set out to study the kinetics of MDSCs in two immunocompetent murine models (i.e. 5TGM1 and MOPC315.BM) and explore the contribution of the two different MDSC subpopulations to diverse disease-promoting aspects in MM. More precisely, we studied the effects of MDSC subsets on MM proliferation and angiogenesis, as reports investigating the role of MDSCs on MM-associated angiogenesis are lacking. Further, it is currently not clear which MDSC subset differentiates into osteoclasts in the context of MM.

## RESULTS

### MDSC kinetics in two murine MM models

**Figure 1:**
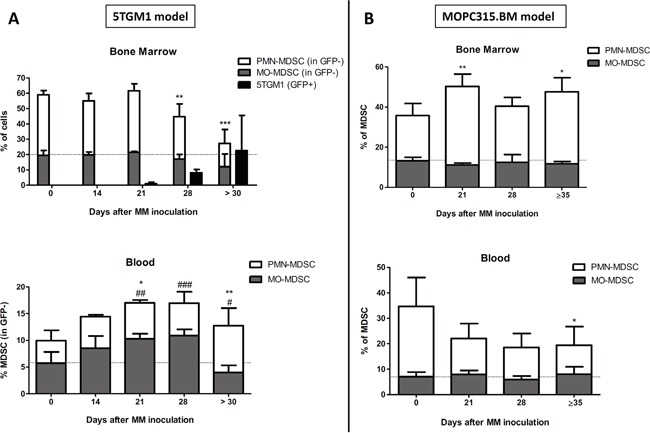
Kinetics of MDSC subpopulations in MM-bearing mice **A.** Percentages (mean ± SD gated on GFP^−^ cells) of granulocytic (PMN-) or monocytic (MO-) MDSC populations in bone marrow or blood of C57BL/KaLwRij mice at day 0 (healthy controls, N=9) and 14 (N=4), 21 (N=4), 28 (N=5) or more than 30 days (N=10) after 5TGM1 inoculation. **B.** Percentages (mean ± SD) of PMN- or MO-MDSC populations in bone marrow or blood of Balb/c mice at day 0 (healthy controls, N=5) and 21 (N=5), 28 (N=5) or 35-38 days (N=6) after MOPC315.BM inoculation. Significant difference compared to healthy controls for PMN-MDSCs (*) or MO-MDSCs (#). *, #p<0.05; **, ##p<0.01; ***, ###p<0.001 (Mann-Whitney test).

We first studied the kinetics of MDSC subpopulations during MM progression in the immunocompetent murine 5TGM1 and MOPC315.BM models using flow cytometry. In the BM, a significant decrease in percentages of PMN-MDSCs and a trend towards decreased MO-MDSC levels was noted in the 5TGM1 model, whereas increased PMN-MDSC levels were observed in the MOPC315.BM model along with MM development (Figure 1A and 1B). In the blood, opposite changes were seen in PMN-MDSC levels, with an expansion in the 5TGM1 model and a decrease in the MOPC315.BM model. Finally, in the 5TGM1 model, MO-MDSCs expanded transiently in the blood, but at the final disease stage their percentage was decreased compared to healthy controls (Figure 1A). Of note, higher levels of both MDSC populations were observed in the BM of naive C57BL/KaLwRij mice compared to naive Balb/c mice (day 0), and the opposite was observed in peripheral blood.

Thus, depending on the MM model, we observed different MDSC kinetics during MM development. However, expansions of PMN-MDSCs were noted in end-stage disease for both models, i.e. in the BM of MOPC315.BM-bearing mice and in the blood of 5TGM1-bearing mice, which was not observed for MO-MDSCs.

### *In vitro* and *in vivo* interactions between myeloma cells and MDSC subpopulations

**Figure 2:**
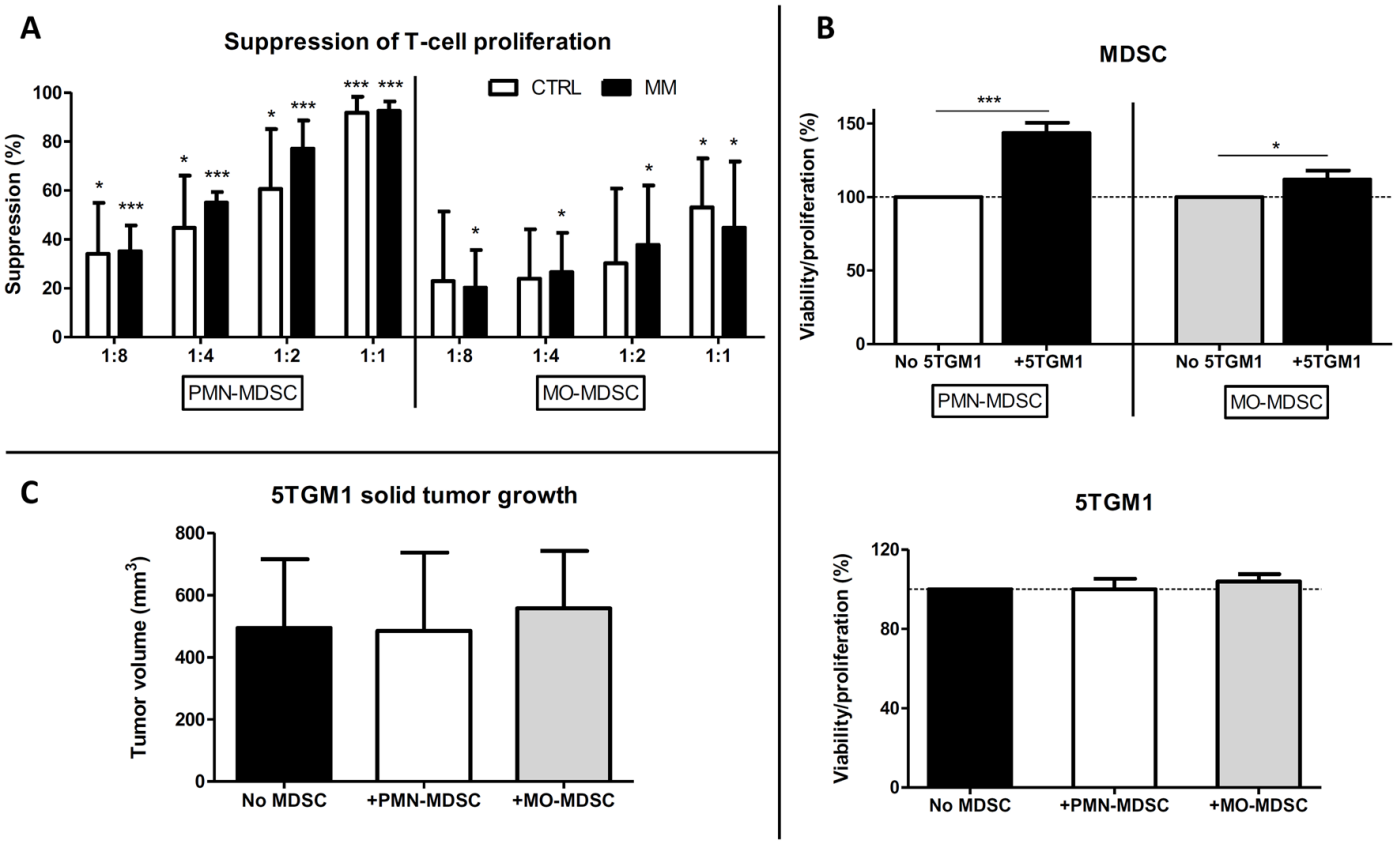
Effects of MDSC subpopulations on T-cell proliferation and interactions with MM cells **A.** Suppression (mean ± SD) of CD3/CD28-induced T-cell proliferation by granulocytic (left) or monocytic (right) MDSCs isolated from 5TGM1-bearing (MM) or healthy (CTRL) C57BL/KaLwRij mice at various MDSC:splenocyte ratios (1:8→1:1). Proliferation was determined using a ^3^H-thymidin incorporation assay. Results represent 3-4 independent experiments and were assessed at least in triplicates within each experiment. *p<0.05; **p<0.01; ***p<0.001 (unpaired Student's T test) when compared to T-cell proliferation without MDSCs (0% suppression). **B.** Percentage of viability/proliferation (mean ± SD), assessed using a MTT assay, of MDSCs from healthy C57BL/KaLwRij mice (top) or 5TGM1 cells (bottom) after 48 hours of contact-independent co-culture in the presence of 5TGM1 cells or MDSC subpopulations isolated from healthy mice, respectively. Results represent 3 independent experiments. *p<0.05; ***p<0.001 (unpaired Student's T test). **C.** 5TGM1 solid tumor volume (mm^3^; mean ± SD) at sacrifice (N=6/group). 5TGM1 cells were subcutaneously injected into the right flank of mice with MDSC subpopulations isolated from 5TGM1-bearing mice (1:1 ratio).

We focused on the 5TGM1 model for further analysis of MDSC subpopulations, as this model has a higher MM infiltration rate than the MOPC315.BM model, which can be easily determined through GFP expression by 5TGM1 cells. Both MDSC subpopulations isolated from the BM of 5TGM1-bearing or healthy mice presented typical morphological characteristics of both subsets (data not shown) and were able to suppress CD3/CD28-induced T-cell proliferation in a dose-dependent manner (Figure 2A). Granulocytic MDSCs were more suppressive than the monocytic population, but no significant difference was seen in the suppressive phenotype of MDSCs isolated from MM-bearing mice or healthy controls.

In order to study the bi-directional interactions between MDSCs and 5TGM1 cells *in vitro*, we performed contact-independent co-cultures of 5TGM1 cells and MDSC subpopulations isolated from healthy C57BL/KaLwRij mice. Using a MTT assay, we assessed the viability of MDSCs (prone to a relatively rapid cell death *in vitro*) and the proliferation of 5TGM1 cells, which survive and rapidly proliferate *in vitro* (thus MTT assay reflects their proliferative activity). The presence of soluble factors secreted by 5TGM1 cells significantly increased the viability of PMN-MDSCs and, to a lesser extent, the viability of MO-MDSCs (Figure 2B). In contrast, the presence of MDSC subpopulations had no effect on the proliferation of 5TGM1 cells *in vitro* (Figure 2B).

The effects of MDSC subpopulations on MM growth *in vivo* were studied on subcutaneous 5TGM1 solid tumor growth. The co-injection of PMN- or MO-MDSCs isolated from MM-bearing mice did not enhance 5TGM1 tumor growth *in vivo* (Figure 2C), even though a small trend towards faster tumor growth was observed at early time points. These results are in accordance with our *in vitro* observations. Furthermore, no significant differences were seen in solid tumor volumes after co-injection with MDSCs from MM-bearing or healthy control mice (data not shown). Finally, MDSC subset percentages and blood vessel counts within these solid tumors were not different between the experimental groups at the point of sacrifice (data not shown).

### 5TGM1 cells instruct granulocytic MDSCs to become pro-angiogenic

**Figure 3:**
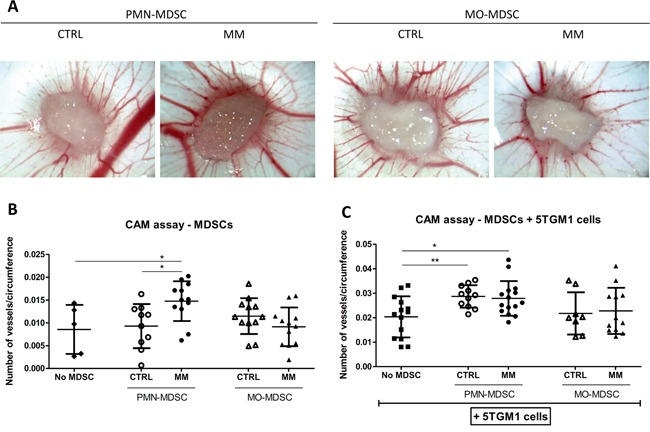
Effects of MDSC subpopulations on angiogenesis in gelatin-sponge CAM assays **A.** Representative pictures (25x magnification) of gelatin-sponge CAM assays in the presence of MDSC subpopulations isolated from healthy (CTRL) or 5TGM1-bearing (MM) C57BL/KaLwRij mice. **B–C.** Quantification of angiogenesis in CAM assays, expressed in the number of blood vessels/sponge circumference (mean ± SD), in the presence of CTRL or MM MDSC subsets. (B) CAM assay in the presence of MDSC subpopulations alone (N=10-12/group). Negative controls (no MDSC) correspond to sponges containing complete medium without cells (N=5). (C) CAM assay in the presence of 5TGM1 cells and MDSC subpopulations. “No MDSC” controls correspond to sponges containing 5TGM1 cells without MDSCs (N=8-15/group). *p<0.05; **p<0.01 (unpaired Student's T test).

We used a gelatin-sponge chick chorioallantoic membrane (CAM) assay in order to directly study the effects of MDSC subpopulations isolated from healthy or myeloma-bearing mice on angiogenesis *in vivo*. The different MDSC subpopulations were implanted on the CAMs alone (Figure 3A and 3B) and in the presence of 5TGM1 cells (Figure 3C). Figure 3A shows representative pictures of CAM assays in the presence of MDSC subpopulations (alone). Interestingly, the quantification of these CAM assays showed a pro-angiogenic effect of PMN-MDSCs isolated from 5TGM1-bearing mice, in contrast to PMN-MDSCs isolated from healthy mice (Figure 3B). Furthermore, in the presence of 5TGM1 cells, the difference between the angiogenic phenotype of PMN-MDSCs isolated from healthy mice or from 5TGM1-bearing mice disappeared (Figure 3C). These results suggest that 5TGM1 cells are able to induce an angiogenic potential in PMN-MDSCs, as PMN-MDSCs obtained from healthy mice are also able to induce an angiogenic response after being co-cultured with 5TGM1 cells.

No significant pro-angiogenic effect of control and MM MO-MDSCs alone or in the presence of 5TGM1 cells was observed in this model (Figure 3B-3C). Of note, a trend towards less angiogenesis on CAM assays was seen in the presence of MO-MDSCs isolated from MM-bearing mice compared to controls.

### Increased expression of pro-angiogenic factors in PMN-MDSCs from MM-bearing mice

We performed an angiogenesis proteome profiler array (R&D Systems) to assess the expression of different angiogenesis-related proteins in MDSC subsets isolated from 5TGM1-bearing mice (MM) compared to MDSCs from healthy mice (CTRL). Expression ratios (MM/CTRL) of the proteins expressed by MDSC subsets are summarized in Table 1. In PMN-MDSCs, several angiogenesis-related proteins are expressed (47 proteins) whereas less of these proteins were detected in MO-MDSCs (24 proteins). Interestingly, an increased expression was noted for the vast majority of these angiogenesis-related proteins (31 proteins out of 47) in PMN-MDSCs isolated from MM-bearing mice compared to controls, among which numerous proteins that have been identified as pro-angiogenic factors in the literature. In contrast, a decreased expression was noted for the vast majority of angiogenesis-related proteins (16 proteins out of 24) in MO-MDSCs from MM-bearing mice, mostly comprising pro-angiogenic factors.

**Table 1:** Expression of angiogenesis-related proteins in BM MDSC subpopulations isolated from 5TMG1 myelomabearing mice (MM) compared to the expression in BM MDSCs from healthy control mice (CTRL)

Analyte	MM/CTRL expression ratio	
	*PMN-MDSCs*	*MO-MDSCs*
ADAMTS1	1.01	/
Amphiregulin	0.90	/
Angiopoietin-1	**1.16**	−0.01
Angiopoietin-3	**1.35**	−0.01
Coagulation factor III	1.01	/
CXCL16	**1.21**	/
Cyr61	**1.49**	/
DLL4	**1.44**	/
DPPIV	0.78	1.06
Endoglin	0.82	/
Endostatin/Collagen18	**1.39**	0.02
Endothelin-1	0.95	0.11
FGF-1	0.78	0.00
FGF-2	**1.23**	0.00
KGF	1.02	/
Fractalkine	**1.75**	/
HB-EGF	**1.32**	/
HGF	0.88	0.79
IGFBP-1	**1.12**	0.06
IGFBP-2	1.02	−0.01
IGFBP-3	**1.78**	−0.01
IL-1alpha	**1.15**	0.01
IL-10	**1.11**	/
IP-10	**1.73**	/
KC (CXCL1)	**1.42**	/
Leptin	**2.13**	/
MCP-1	**1.50**	**1.96**
MIP-1alpha (CCL3)	**2.36**	0.80
MMP-8	0.96	−0.01
MMP-9	**1.24**	0.77
NOV	1.04	/
Osteopontin	1.04	**1.53**
PD-ECGF	**2.66**	/
PDGF-AA	**1.57**	/
PDGF-AB/BB	**1.46**	/
Pentraxin-3	**1.42**	1.05
Platelet factor 4	1.05	**1.74**
PlGF-2	**1.12**	0.42
Prolactin	**1.65**	/
Proliferin	0.83	/
SDF-1	0.78	**3.81**
Serpin E1	**2.03**	/
Serpin F1	**3.78**	/
Thrombospondin-2	**3.07**	/
TIMP-1	**1.95**	**1.14**
TIMP-4	**3.29**	0.29
VEGF-B	**1.43**	**3.06**

[/ : no detectable expression; **bold type**: increased expression (ratio ≥ 1.1) in MDSCs from MM-bearing mice].

These results are in accordance with a pro-angiogenic role of PMN-MDSCs in the context of MM through the expression and up-regulation of numerous angiogenesis-related factors.

### *In vivo* targeting of MDSCs results in a decreased myeloma-induced angiogenesis

Within the BM of 5TGM1-bearing mice that were treated with anti-Gr1 antibody, De Veirman *et al* described reduced levels of MDSCs, with a preferential depletion of PMN-MDSCs, leading to a reduced tumor load in the BM of these mice [19]. As a preferential depletion of the granulocytic MDSC fraction has been shown in the BM of these mice, we analyzed the microvessel density (MVD) in BM sections from these mice and found that it was significantly lower in the anti-Gr1-treated 5TGM1-bearing mice compared to vehicle-treated 5TGM1-bearing mice, i.e. 23 ± 3.5 versus 33.6 ± 3.3 vessels/field (p = 0.004), respectively (Supplementary Figure 1). These *in vivo* results reinforce our previous results that suggested a pro-angiogenic role for PMN-MDSCs.

### MO-MDSCs are able to differentiate into osteoclasts

**Figure 4:**
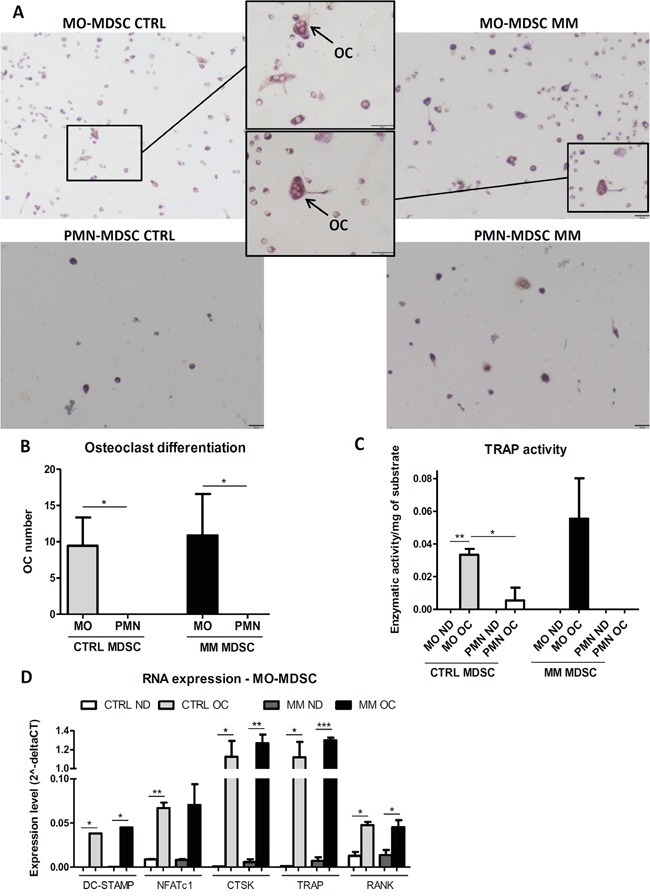
Differentiation of MDSC subpopulations into osteoclasts Osteoclast (OC) differentiation cultures were performed on MDSC subpopulations isolated from healthy (CTRL) or 5TGM1-bearing (MM) C57BL/KaLwRij mice (ND = non-differentiated control cultures). **A.** Representative pictures of TRAP staining on OC differentiation cultures (black bars = 50 μm). **B.** Total osteoclast number (mean ± SD) counted in 3 fields/well after OC differentiation. OCs are defined as TRAP^+^-stained cells with multiple nuclei (≥3). **C.** TRAP activity (mean ± SD of enzymatic activity/mg of substrate) measured in culture supernatants. **D.** RNA expression levels (mean ± SD of 2^−dCT^) of different OC-related factors in MO-MDSCs at the end of the cultures. All the results represent at least 2-3 independent experiments. Within experiments, triplicates were performed for each condition. *p<0.05; **p<0.01, ***p<0.001 (unpaired Student's T test).

Finally, we determined whether both MDSC subpopulations or, in contrast, only one of the two heterogeneous MDSC subsets contains osteoclast (OC) precursors. MO-MDSCs were able to differentiate into Tartrate-Resistant Acid Phosphatase (TRAP)^+^ multinucleated OCs *in vitro*, in contrast to PMN-MDSCs (Figure 4A-4B). This was further corroborated by the detection of specific TRAP activity in the supernatant of MO-MDSC-derived OC cultures (Figure 4C). RNA expression analysis showed a significant increase of factors that are crucial for OC differentiation and/or activity in OC-differentiated MO-MDSCs, i.e. dendritic cell-specific transmembrane protein (DC-STAMP), nuclear factor of activated T cells c1 (NFATc1), receptor activator of nuclear factor κ-B (RANK), cathepsin K (CTSK) and TRAP, confirming the OC phenotype of the differentiated MO-MDSCs (Figure 4D).

However, no significant difference was noted between MM and CTRL MO-MDSCs in their ability to differentiate into OCs, in specific TRAP activity or in RNA expression of OC-related factors (Figure 4A-4D).

## DISCUSSION

In this study, we focused on the relative contribution of granulocytic and monocytic MDSC subsets to different aspects of MM disease in murine models. Previous reports showed different results regarding the expansion of granulocytic or monocytic MDSC subpopulations in MM. In the current study, we observed opposite PMN-MDSC kinetics in two murine models, i.e. the 5TGM1 and MOPC315.BM models, and different MDSC levels in naive C57BL/KaLwRij compared to Balb/c mice. Schmid *et al* reported similar results, showing that the genetic background of C57BL/6 or Balb/c mice influenced the prevalence of MDSC subsets in the BM of naive mice, as well as their differentiation and function [20]. However, in both murine MM models we observed an expansion of the granulocytic MDSC fraction in different compartments of end-stage diseased animals, in contrast to the monocytic fraction. Our results suggest that PMN-MDSCs could be mobilized from the BM into peripheral blood in the 5TGM1 model, whereas in the MOPC315.BM model these cells remain inside the BM. A recent report showed similar data, with a transient MO-MDSC expansion and a final increase in PMN-MDSCs (end-stage disease) in peripheral blood of C57BL/ KaLwRij mice bearing the 5TGM1-parental 5T33 myeloma [19]. However, in our study some intermediate time points included a relatively small number of animals (N=4) and need confirmation in future studies. In the MOPC315.BM model, a strong downregulation of all hematopoietic precursor cells except those from myeloid lineage was reported early upon MM infiltration in the BM [21]. Thus, we can not exclude that the observed increases in MDSC percentages are partially caused by a reduction in total BM cells. Both MDSC subsets isolated from 5TGM1 myeloma-bearing mice were immunosuppressive. The granulocytic fraction was more suppressive than the monocytic, as reported previously for MDSCs isolated from the BM of MM patients [15]. However, no difference was seen in the suppressive potential of MDSCs isolated from MM-bearing mice or healthy controls. In contrast to studies reporting that immature myeloid cells from naive mice are not immunosuppressive [14, 22], our results are in accordance with a report from Forghani *et al* who observed a comparable suppressive capacity for MDSCs isolated from the BM of tumor-free or mammary tumor-bearing mice, suggesting a physiological role of MDSCs in the control of T-cell proliferation and activation in the BM microenvironment [23]. Similarly, no significant difference was reported between the suppressive potential of MDSCs isolated from MM patients or healthy donors [16].

MDSC subsets did not influence MM proliferation *in vitro* or *in vivo*. Of note, no significant differences in MDSC levels were seen in 5TGM1 solid tumors at the moment of sacrifice. MDSCs might have differentiated into mature myeloid cells, migrated towards other sites or have been prone to cell death. In addition, endogenous MDSCs from tumor-bearing mice could have been recruited towards the tumor site, annulling the initial differences in MDSC levels between groups. In future studies, it will be of interest to label co-injected MDSCs to distinguish them from endogenous MDSCs. In contrast to these findings, soluble factors secreted by MM cells increased the viability of both MDSC subsets, especially PMN-MDSCs, *in vitro*. Similar observations were made by De Veirman *et al* who showed that conditioned medium from 5T33MM cells increased the viability of total MDSCs [19].

We describe for the first time a pro-angiogenic role of MDSCs in the context of MM. Interestingly, this pro-angiogenic role is exerted by granulocytic MDSCs, in contrast to previous studies that described a pro-angiogenic role of total MDSCs or monocytic MDSCs in other cancers [8–11]. The angiogenic phenotype of PMN-MDSCs is dependent on the presence of MM cells *in vivo* or “*ex vivo*” (on CAM assays), suggesting that MM cells directly instruct PMN-MDSCs to become pro-angiogenic. A possible mechanism for the pro-angiogenic potential of PMN-MDSCs lies in the expression of numerous angiogenesis-related factors by these cells, among which a vast majority is up-regulated in granulocytic MDSCs isolated from MM-bearing mice compared to control MDSCs. Finally, our findings are reinforced by *in vivo* results, as we show a reduced blood vessel density in the BM of MM-bearing mice when less PMN-MDSCs are present. Of note, anti-Gr1-treated mice also display decreased levels of 5TGM1 infiltration within BM (4.8-fold reduction compared to vehicle-treated mice). Thus, we cannot completely exclude an effect of reduced MM infiltration on the observed decrease of angiogenesis. In contrast, MO-MDSCs had no pro-angiogenic effect, expressed less angiogenesis-related factors than PMN-MDSCs, and the majority of these factors were expressed to a lesser extent in MO-MDSCs isolated from myeloma-bearing mice compared to control MDSCs. The angiogenic potential of total MDSCs in tumor-bearing hosts was shown by others to be associated with the production of MMP-9 [8]. Here, we show that MMP-9 protein expression is slightly increased in granulocytic and decreased in monocytic MM MDSCs.

Even though MO-MDSCs do not seem to play a role in MM-related angiogenesis, this subpopulation may be implicated in MM-associated osteolytic bone disease, as we identified these cells as osteoclast precursors. Zhuang *et al* [18] demonstrated that MDSCs from 5TGM1-bearing mice could differentiate into osteoclasts *in vitro* and *in vivo*. However, they did not identify the specific MDSC subpopulation that contains OC progenitors, as they only considered total MDSCs (CD11b^+^Gr1^+^). Despite an important heterogeneity within both MDSC subpopulations, we showed that the PMN-MDSC subset does not contain any cellular fraction able to differentiate into OCs. In contrast to Zhuang *et al*, we did not see significant differences in OC differentiation from MO-MDSCs isolated from MM-bearing or control mice. Similarly, reports on OC differentiation in breast cancer bone metastasis only considered total MDSCs and not individual subsets [24, 25].

Targeting of total MDSCs using anti-Gr1 antibodies or 5-fluorouracil in the murine 5TGM1 or 5T33MM models, respectively, showed promising results with a significant reduction of MM burden [19]. However, insight into the mechanisms by which this occurs is lacking and a direct effect of these strategies on myeloma cells should be investigated. In future studies, it would be interesting to target more specifically the granulocytic or monocytic MDSC subpopulations in myeloma, with the aim to reduce MM-related immune suppression, angiogenesis or osteolytic bone disease, and thus reduce MM progression. Notably, blocking of the programmed cell death (PD) 1 receptor and its ligand, PD-L1, which respectively present increased expression levels on immune effector cells and MDSCs in MM patients, inhibited MDSC-mediated immune suppression. In addition, as MM cells express high levels of PD-L1, blockade of this axis also directly inhibits stromal cell-induced MM growth, suggesting a therapeutical interest of targeting the PD-1/PD-L1 axis in MM [26].

In conclusion, we show a preferential expansion of granulocytic MDSCs in two different murine MM models, even though the expansion occurs in different compartments. Furthermore, the granulocytic subset has a higher immunosuppressive capacity than MO-MDSCs. We describe for the first time a pro-angiogenic role of granulocytic MDSCs in the context of MM. Our results suggest that myeloma cells instruct PMN-MDSCs to become pro-angiogenic by inducing changes in the expression of multiple angioregulatory factors by PMN-MDSCs. Thus, we determined that the granulocytic MDSC subpopulation plays a major role in different myeloma-promoting disease aspects and we showed that the monocytic MDSC subset contains osteoclast precursors, demonstrating that MDSC subpopulations play diverging roles in MM.

## MATERIALS AND METHODS

### Mice

C57BL/KaLwRijHsd mice were purchased from Harlan laboratories B.V. (Horst, Netherlands) and Balb/cJ mice from Jackson Laboratory (Bar Harbor, USA). Both strains were bred at the animal facility of our institute and mice were used for experiments when they were between 10- to 14-wk-old. Animal welfare was assessed at least once per day, and all efforts were made to minimize animal suffering during the experiments. Experimental procedures used in this investigation were approved by the Institutional Animal Care and Use Ethics Committee of the University of Liège (Belgium).

### Myeloma cell lines and models

Two murine myeloma cell lines resulting in MM disease after intravenous (i.v.) injection to syngeneic mice were used. The selection of the Balb/c-derived MOPC315.BM cell line, kindly provided by B. Bogen (University of Oslo, Oslo, Norway), and the labeling with luciferase gene have been described previously [27, 28]. The establishment of the 5TGM1 cell line and eGPF-transfected 5TGM1 cells, originating from C57BL/KaLwRij mice and kindly provided by G.R. Mundy and C.M. Edwards (Vanderbilt University, Nashville, TN, USA), has been described previously [29, 30]. Both cell lines were maintained in culture at 37°C in 5% CO_2_, using RPMI 1640 medium for MOPC315.BM cells or Dulbecco's Modified Eagle Medium for 5TGM1 cells, supplemented with 10% heat-inactivated fetal bovine serum (FBS) and Penicillin/Streptomycin (100 U/ml) (=complete medium). All cell culture products were purchased from Lonza (Verviers, Belgium).

*In vivo* experiments for both MM models (i.v. injections) and monitoring of MOPC315.BM tumor development by bioluminescence were performed as previously described [31, 32]. For 5TGM1 solid tumor growth, 5TGM1 cells were suspended in phosphate buffered saline (PBS) containing 1 mg/ml of matrigel and were injected subcutaneously (2.5×10^5^ cells/200μl/mouse) into the right flank of C57BL/KaLwRij mice. For these solid tumor experiments, mice in all groups were sacrificed when tumors exceeded 1 cm or when skin ulcerations appeared (4 mice/group were sacrificed at day 18 after injection, all the other mice at day 22).

### Isolation of MDSC subpopulations

BM from tibias, femurs and spine was harvested from 5TGM1-bearing or healthy C57BL/KaLwRij mice and homogenized in sterile culture medium. Red blood cells were lysed using a RBC lysis buffer (eBioscience, San Diego, USA) and cells were washed with PBS and filtered through a 70 μM nylon membrane to obtain final cell suspensions. More than 90% of CD11b^+^ BM cells are Ly6G^+^ or Ly6C^+^ MDSCs. MDSC subpopulations were isolated from BM cells using magnetic cell sorting. Granulocytic MDSCs were isolated on a LS column through a positive selection for Ly6G using the mouse anti-Ly6G microbead kit (Miltenyi Biotec, Bergisch Gladbach, Germany) according to the manufacturer's instructions. The Ly6G^−^ fraction was further depleted for residual Ly6G^+^ cells using a LD column, followed by a positive selection for CD11b using a LS column and mouse/human CD11b microbeads (Miltenyi Biotec) to obtain the monocytic MDSC fraction. Purity was assessed for each fraction using flow cytometry.

### Suppression of T-cell proliferation

Spleens were harvested from C57BL/KaLwRij mice and cell suspensions were obtained as described above. Splenocytes (1×10^5^/well) were cultured in 96-well plates using complete RPMI 1640 medium containing 5 μM of 2-mercaptoethanol (Gibco by Life Technologies, Gent, Belgium). MDSC subsets were added (final culture volume 200 μl/well) at different MDSC:splenocyte ratios (1:8; 1:4; 1:2; 1:1) and T-cell proliferation was induced by adding 1 μl/well of mouse T-activator CD3/CD28 dynabeads (Gibco). Proliferation was assessed after 72 hours of culture by adding 0.33 μCi of [methyl-^3^H] thymidin (Perkin Elmer, Waltham, USA) to each well for the last 18 hours of culture. DNA was harvested on Multiscreen Harvest Plates (Millipore, Carrigtwohill, Ireland) using Filter Mate Harvester (Perkin Elmer). Plates were dried for 3-4 hours before adding 25 μl/well of Microscint-O (Perkin Elmer) and measuring radioactivity (c.p.m.) with a TopCount NXT Microplate Scintillation counter (Perkin Elmer). Suppression was calculated as follows: Suppression (%) = [1 − (Proliferation with MDSCs/ proliferation without MDSCs)] x 100.

### MDSC and 5TGM1 viability/proliferation *in vitro*

MDSC subpopulations were isolated from healthy C57BL/KaLwRij mice as described above. Contact-independent co-cultures of MDSCs (2×10^6^) and 5TGM1 cells (5×10^5^) were performed using 0.4 μM pore size transwell inserts (ThinCert, Greiner Bio-one, Frickenhausen, Germany). After 48 hours, MDSCs and 5TGM1 cells were harvested and viability/proliferation was assessed using the cell proliferation kit I (MTT) from Roche Applied Science (Mannheim, Germany) following the manufacturer's instructions. Absorbance at 570 nm was measured using a Wallac 1420 Victor2 microplate reader (Perkin Elmer). Viability/proliferation was expressed in percentage relative to MDSCs or 5TGM1 cells cultured alone (=100%).

### Flow cytometry

Cell suspensions were obtained from spleen, bone marrow or peripheral blood as described above. Extracellular staining was performed in PBS containing 3% FBS and antibodies were incubated for 30 min at 4°C. MDSC levels were determined by gating on CD11b^+^Ly6G^+^Ly6C^int/low^ cells (PMN-MDSCs) or CD11b^+^Ly6G^−^Ly6C^+^ cells (MO-MDSCs). Anti-mouse CD11b/APC (M1/70) was purchased from eBioscience (San Diego, USA) and Ly-6G/Pacific Blue (1A8) and Ly-6C/PECy7 (HK1.4) were purchased from Biolegend (San Diego, USA).

### Chick chorioallantoic membrane (CAM) assay

Chicken eggs were disinfected and 8-9 ml of albumen were aspirated in order to dissociate the CAM from the egg shell membrane 3 days after fertilization. An observation window was carefully cut into the shell, followed by re-injection of 4-5 ml of albumen below the CAM. The window was covered during further incubations in an egg incubator at 37°C and 80% humidity. We adapted a gelatin-sponge CAM assay from previous reports [33–35] using Gelfoam gelatin sponges (Pharmacia and Upjohn Company, Kalamazoo, USA), cut into 1-2 mm^3^ cubes, as support for non-adherent cells as previously described for macrophages [36]. The sponge was implanted on the CAM on day 9 post-fertilization and MDSCs (2.5×10^5^ cells) and/or 5TGM1 cells (5×10^4^ cells) were suspended in complete medium (5 μl) and placed on the sponge. At day 13 or 16, respectively, eggs incubated with MDSCs alone or with MDSCs in the presence of 5TGM1 cells were placed during 30 minutes at −20°C and CAMs were fixed *in ovo* using a 4% paraformaldehyde solution. CAM portions containing the sponges were excised, observed using a MZ75 stereomicroscope (Leica Microsystems, Wetzlar, Germany) at 12.5x or 25x magnification and pictures were taken using the Leica Acquire Software (Leica Microsystems). Angiogenesis was quantified by a person blinded to the identity of samples, by counting the number of sponge-directed blood vessels on the CAM-sponge interface. This number was divided by the circumference of the corresponding sponge, calculated using the length and width of the sponge [circumference = Π x √(2x((length/2)^2^ + (width/2)^2^))] measured using the ImageJ software (National Institutes of Health, USA).

### Angiogenesis-related protein array

Mouse angiogenesis proteome profiler array (R&D Systems, Minneapolis, USA) was performed on cell lysates from MDSC subpopulations isolated from 5TGM1 myeloma-bearing (MM) or healthy C57BL/KaLwRij control mice (CTRL) following the manufacturer's instructions. Signals were quantified using the QuantityOne 1-D Analysis Software (Biorad, Hercules, USA). Mean values of background signals (negative control spots) were substracted from mean values of analyte spots for each membrane, and MM/CTRL expression ratio was determined for proteins expressed by MDSCs.

### *In vivo* treatment with anti-Gr1 antibody

5TGM1 cells were intravenously inoculated to syngeneic mice (1 × 10^6^ cells/mouse). One day later, treatment was started with 200 μg/mouse of anti-Gr1 antibody (RB6–8C5 purchased from BioXCell, West Lebanon, NH) every two days. When first mice showed signs of disease, all mice were sacrificed. CD31 immunostaining and subsequent microvessel density (MVD) quantification were performed on BM sections from these mice as described previously [37].

### Osteoclast differentiation

MDSC subsets isolated from 5TGM1 myeloma-bearing or healthy C57BL/KaLwRij control mice were suspended in differentiation medium, i.e. α-Minimum Essential Medium supplemented with 10% heat-inactivated FBS, Penicillin/Streptomycin (100 U/ml), Glutamine (2mM), 20 ng/ml M-CSF (Peprotech, Rocky Hill, USA) and 100 ng/ml receptor activator of nuclear factor κ-B ligand (RANKL, Peprotech). Using 48-well plates (Greiner Bio-One), 1.25 × 10^5^ cells were plated into each well and cultured for 5-6 days at 37°C and 5% CO2. In non-differentiated (ND) control wells, no RANKL was added to the medium. Osteoclast staining was performed using Leukocyte Tartrate-Resistant Acid Phosphatase (TRAP) kit (Sigma-Aldrich, St. Louis, USA) following the manufacturer's instructions. For the quantification of specific TRAP activity, culture supernatants (10 μl) were added to a p-nitrophenylphosphate (7.6 mM) sodium acetate-tartrate solution (Sigma-Aldrich) and incubated 1h at 37°C. The reaction was stopped by the addition of NaOH and absorbance of the yellow enzymatic product p-nitrophenyl (pNP) was measured at 405 nm. Absorbance values were compared to a pNP standard curve and specific enzymatic activity was calculated [38].

### RNA expression

RNA was isolated using the RNeasy Mini kit (Qiagen, Venlo, Netherlands) according to the manufacturer's instructions. Genomic DNA was removed using recombinant RNase-free DNaseI (Roche, Vilvoorde, Belgium). cDNA synthesis was performed on RNA (100 ng) with random hexamer primers using the Transcriptor First Strand cDNA Synthesis Kit (Roche). Quantitative real-time PCR (qPCR) was performed with KAPA SYBR FAST qPCR kit (KAPA biosystems, Wilmington, USA) on Light cycler 480 (Roche). All primers were synthesized by Integrated DNA Technologies (Leuven, Belgium) and are listed in Supplementary Table 1. RNA expression levels (2^−dCT^) were calculated using actin and β-2 microglobulin reference genes.

### Statistics

Statistical significance between murine experimental groups was determined using the Mann-Whitney test calculated with the Prism Software (Graph Pad Software, San Diego, CA). An unpaired Student's T-Test performed with Excel software was used to determine statistical significance for other experiments.

## SUPPLEMENTARY FIGURE AND TABLE



## References

[1] Caers J, Vande broek I, De Raeve H, Michaux L, Trullemans F, Schots R, Van Camp B, Vanderkerken K (2008). Multiple myeloma--an update on diagnosis and treatment. European journal of haematology.

[2] Otjacques E, Binsfeld M, Noel A, Beguin Y, Cataldo D, Caers J (2011). Biological aspects of angiogenesis in multiple myeloma. International journal of hematology.

[3] Binsfeld M, Fostier K, Muller J, Baron F, Schots R, Beguin Y, Heusschen R, Caers J (2014). Cellular immunotherapy in multiple myeloma: lessons from preclinical models. Biochimica et biophysica acta.

[4] Gabrilovich DI, Nagaraj S (2009). Myeloid-derived suppressor cells as regulators of the immune system. Nature reviews Immunology.

[5] Movahedi K, Guilliams M, Van den Bossche J, Van den Bergh R, Gysemans C, Beschin A, De Baetselier P, Van Ginderachter JA (2008). Identification of discrete tumor-induced myeloid-derived suppressor cell subpopulations with distinct T cell-suppressive activity. Blood.

[6] Youn JI, Nagaraj S, Collazo M, Gabrilovich DI (2008). Subsets of myeloid-derived suppressor cells in tumor-bearing mice. Journal of immunology.

[7] Ye XZ, Yu SC, Bian XW (2010). Contribution of myeloid-derived suppressor cells to tumor-induced immune suppression, angiogenesis, invasion and metastasis. Journal of genetics and genomics.

[8] Yang L, DeBusk LM, Fukuda K, Fingleton B, Green-Jarvis B, Shyr Y, Matrisian LM, Carbone DP, Lin PC (2004). Expansion of myeloid immune suppressor Gr+CD11b+ cells in tumor-bearing host directly promotes tumor angiogenesis. Cancer cell.

[9] Okazaki T, Ebihara S, Asada M, Kanda A, Sasaki H, Yamaya M (2006). Granulocyte colony-stimulating factor promotes tumor angiogenesis via increasing circulating endothelial progenitor cells and Gr1+CD11b+ cells in cancer animal models. International immunology.

[10] Mucha J, Majchrzak K, Taciak B, Hellmen E, Krol M (2014). MDSCs mediate angiogenesis and predispose canine mammary tumor cells for metastasis via IL-28/IL-28RA (IFN-lambda) signaling. PloS one.

[11] Priceman SJ, Sung JL, Shaposhnik Z, Burton JB, Torres-Collado AX, Moughon DL, Johnson M, Lusis AJ, Cohen DA, Iruela-Arispe ML, Wu L (2010). Targeting distinct tumor-infiltrating myeloid cells by inhibiting CSF-1 receptor: combating tumor evasion of antiangiogenic therapy. Blood.

[12] Greten TF, Manns MP, Korangy F (2011). Myeloid derived suppressor cells in human diseases. International immunopharmacology.

[13] Brimnes MK, Vangsted AJ, Knudsen LM, Gimsing P, Gang AO, Johnsen HE, Svane IM (2010). Increased level of both CD4+FOXP3+ regulatory T cells and CD14+HLA-DR(-)/low myeloid-derived suppressor cells and decreased level of dendritic cells in patients with multiple myeloma. Scandinavian journal of immunology.

[14] Ramachandran IR, Martner A, Pisklakova A, Condamine T, Chase T, Vogl T, Roth J, Gabrilovich D, Nefedova Y (2013). Myeloid-derived suppressor cells regulate growth of multiple myeloma by inhibiting T cells in bone marrow. Journal of immunology.

[15] Gorgun GT, Whitehill G, Anderson JL, Hideshima T, Maguire C, Laubach J, Raje N, Munshi NC, Richardson PG, Anderson KC (2013). Tumor-promoting immune-suppressive myeloid-derived suppressor cells in the multiple myeloma microenvironment in humans. Blood.

[16] Favaloro J, Liyadipitiya T, Brown R, Yang S, Suen H, Woodland N, Nassif N, Hart D, Fromm P, Weatherburn C, Gibson J, Ho PJ, Joshua D (2014). Myeloid derived suppressor cells are numerically, functionally and phenotypically different in patients with multiple myeloma. Leukemia & lymphoma.

[17] Van Valckenborgh E, Schouppe E, Movahedi K, De Bruyne E, Menu E, De Baetselier P, Vanderkerken K, Van Ginderachter JA (2012). Multiple myeloma induces the immunosuppressive capacity of distinct myeloid-derived suppressor cell subpopulations in the bone marrow. Leukemia.

[18] Zhuang J, Zhang J, Lwin ST, Edwards JR, Edwards CM, Mundy GR, Yang X (2012). Osteoclasts in multiple myeloma are derived from Gr-1+CD11b+myeloid-derived suppressor cells. PloS one.

[19] De Veirman K, Van Ginderachter JA, Lub S, De Beule N, Thielemans K, Bautmans I, Oyajobi BO, De Bruyne E, Menu E, Lemaire M, Van Riet I, Vanderkerken K, Van Valckenborgh E (2015). Multiple myeloma induces Mcl-1 expression and survival of myeloid-derived suppressor cells. Oncotarget.

[20] Schmid M, Zimara N, Wege AK, Ritter U (2014). Myeloid-derived suppressor cell functionality and interaction with Leishmania major parasites differ in C57BL/6 and BALB/c mice. European journal of immunology.

[21] Matthes T, Manfroi B, Zeller A, Dunand-Sauthier I, Bogen B, Huard B (2015). Autocrine amplification of immature myeloid cells by IL-6 in multiple myeloma-infiltrated bone marrow. Leukemia.

[22] Kusmartsev S, Nagaraj S, Gabrilovich DI (2005). Tumor-associated CD8+ T cell tolerance induced by bone marrow-derived immature myeloid cells. Journal of immunology.

[23] Forghani P, Harris W, Giver CR, Mirshafiey A, Galipeau J, Waller EK (2013). Properties of immature myeloid progenitors with nitric-oxide-dependent immunosuppressive activity isolated from bone marrow of tumor-free mice. PloS one.

[24] Danilin S, Merkel AR, Johnson JR, Johnson RW, Edwards JR, Sterling JA (2012). Myeloid-derived suppressor cells expand during breast cancer progression and promote tumor-induced bone destruction. Oncoimmunology.

[25] Sawant A, Deshane J, Jules J, Lee CM, Harris BA, Feng X, Ponnazhagan S (2013). Myeloid-derived suppressor cells function as novel osteoclast progenitors enhancing bone loss in breast cancer. Cancer research.

[26] Gorgun G, Samur MK, Cowens KB, Paula S, Bianchi G, Anderson JE, White RE, Singh A, Ohguchi H, Suzuki R, Kikuchi S, Harada T, Hideshima T, Tai YT, Laubach JP, Raje N (2015). Lenalidomide Enhances Immune Checkpoint Blockade-Induced Immune Response in Multiple Myeloma. Clinical cancer research.

[27] Lauritzsen GF, Bogen B (1993). The role of idiotype-specific, CD4+ T cells in tumor resistance against major histocompatibility complex class II molecule negative plasmacytoma cells. Cellular immunology.

[28] Hofgaard PO, Jodal HC, Bommert K, Huard B, Caers J, Carlsen H, Schwarzer R, Schunemann N, Jundt F, Lindeberg MM, Bogen B (2012). A novel mouse model for multiple myeloma (MOPC315. BM) that allows noninvasive spatiotemporal detection of osteolytic disease. PloS one.

[29] Garrett IR, Dallas S, Radl J, Mundy GR (1997). A murine model of human myeloma bone disease. Bone.

[30] Oyajobi BO, Munoz S, Kakonen R, Williams PJ, Gupta A, Wideman CL, Story B, Grubbs B, Armstrong A, Dougall WC, Garrett IR, Mundy GR (2007). Detection of myeloma in skeleton of mice by whole-body optical fluorescence imaging. Molecular cancer therapeutics.

[31] Binsfeld M, Beguin Y, Belle L, Otjacques E, Hannon M, Briquet A, Heusschen R, Drion P, Zilberberg J, Bogen B, Baron F, Caers J (2014). Establishment of a murine graft-versus-myeloma model using allogeneic stem cell transplantation. PloS one.

[32] Otjacques E, Binsfeld M, Rocks N, Blacher S, Vanderkerken K, Noel A, Beguin Y, Cataldo D, Caers J (2013). Mithramycin exerts an anti-myeloma effect and displays anti-angiogenic effects through up-regulation of anti-angiogenic factors. PloS one.

[33] Ribatti D, Gualandris A, Bastaki M, Vacca A, Iurlaro M, Roncali L, Presta M (1997). New model for the study of angiogenesis and antiangiogenesis in the chick embryo chorioallantoic membrane: the gelatin sponge/chorioallantoic membrane assay. Journal of vascular research.

[34] Ribatti D, Nico B, Vacca A, Presta M (2006). The gelatin sponge-chorioallantoic membrane assay. Nature protocols.

[35] Movahedi B, Gysemans C, Jacobs-Tulleneers-Thevissen D, Mathieu C, Pipeleers D (2008). Pancreatic duct cells in human islet cell preparations are a source of angiogenic cytokines interleukin-8 and vascular endothelial growth factor. Diabetes.

[36] Movahedi K, Laoui D, Gysemans C, Baeten M, Stange G, Van den Bossche J, Mack M, Pipeleers D, In't Veld P, De Baetselier P, Van Ginderachter JA (2010). Different tumor microenvironments contain functionally distinct subsets of macrophages derived from Ly6C(high) monocytes. Cancer research.

[37] Van Valckenborgh E, De Raeve H, Devy L, Blacher S, Munaut C, Noel A, Van Marck E, Van Riet I, Van Camp B, Vanderkerken K (2002). Murine 5T multiple myeloma cells induce angiogenesis *in vitro* and *in vivo*. British journal of cancer.

[38] Muller M, Torger B, Wehrum D, Vehlow D, Urban B, Woltmann B, Hempel U (2015). Drug delivery and cell interaction of adhesive poly(ethyleneimine)/sulfated polysaccharide complex particle films. Biointerphases.

