# Supplementary material for: Granulocytic myeloid-derived suppressor cells promote angiogenesis in the context of multiple myeloma
Source: Oncotarget. 2016 May 10;7(25):37931–43. doi: 10.18632/oncotarget.9270 (PMC5122361; doi:10.18632/oncotarget.9270)
Supplement: Supplementary file 1 [file oncotarget-07-37931-s001.pdf]

Granulocytic myeloid-derived suppressor cells promote angiogenesis in the context of multiple myeloma

SUPPLEMENTARY FIGURE AND TABLE

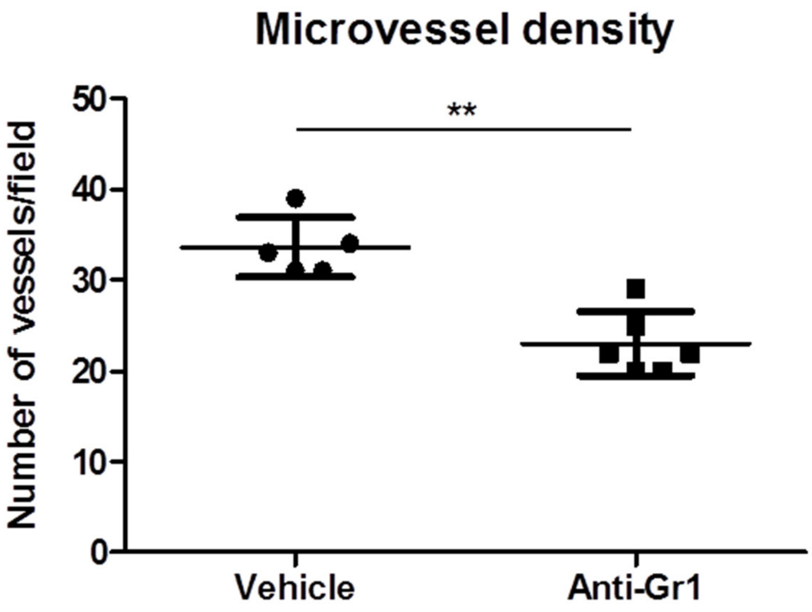

Supplementary Figure S1: Effect of *in vivo* PMN-MDSC-targeting on angiogenesis. Microvessel density (MVD) was assessed by CD31 immunostaining and subsequent quantification of blood vessels (mean number of blood vessels/field ± SD) on BM sections from vehicle-treated or anti-Gr1-treated 5TGM1-bearing mice (N = 5 or 6, respectively). **p<0.01 (Mann-Whitney test).

Supplementary Table S1: qPCR primer list

Analyte (murine)	Forward (F) – Reverse (R) primer	Sequence
DC-STAMP	F	TTGCCGCTGTGGACTATCTG
	R	GAATGCAGCTCGGTTCAAAC
NFATc1	F	TGAGGCTGGTCTTCCGAGTT
	R	CGCTGGGAACACTCGATAGG
CTSK	F	CAGCAGAGGTGTGTACTATG
	R	GCGTTGTTCTTATTCCGAGC
TRAP	F	TCCTGGCTCAAAAAGCAGTT
	R	ACATAGCCCACACCGTTCTC
RANK	F	CGAGGAAGATTCCCACAGAG
	R	CAGTGAAGTCACAGCCCTCA
